# Epidemiology and risk factors for mortality in critically ill patients with pancreatic infection

**DOI:** 10.1016/j.jointm.2023.06.004

**Published:** 2023-08-30

**Authors:** Marie Dejonckheere, Massimo Antonelli, Kostoula Arvaniti, Koen Blot, Ben CreaghBrown, Dylan W. de Lange, Jan De Waele, Mieke Deschepper, Yalim Dikmen, George Dimopoulos, Christian Eckmann, Guy Francois, Massimo Girardis, Despoina Koulenti, Sonia Labeau, Jeffrey Lipman, Fernando Lipovestky, Emilio Maseda, Philippe Montravers, Adam Mikstacki, JoseArtur Paiva, Cecilia Pereyra, Jordi Rello, JeanFrancois Timsit, Dirk Vogelaers, Stijn Blot, the Abdominal Sepsis Study (AbSeS) group on behalf of the Trials Group of the European Society of Intensive Care Medicine

**Affiliations:** 1 Department of Internal Medicine and Pediatrics, Faculty of Medicine and Health Sciences, Ghent University, Ghent, Belgium; 2 Department of Anesthesiology, Intensive Care and Emergency Medicine, Fondazione Policlinico Universitario A. Gemelli IRCCS, Rome, Italy; 3 Università Cattolica del Sacro Cuore, Rome, Italy; 4 Intensive Care Unit, Papageorgiou University Affiliated Hospital, Thessaloníki, Greece; 5 Department of Epidemiology and Public Health, Sciensano, Ixelles, Belgium; 6 Surrey Perioperative Anaesthetic Critical Care Collaborative Research Group (SPACeR), Royal Surrey County Hospital, Guildford, UK; 7 Department of Clinical and Experimental Medicine, University of Surrey, Guildford, UK; 8 Department of Intensive Care Medicine, University Medical Center Utrecht, University Utrecht, Utrecht, the Netherlands; 9 Department of Intensive Care Medicine, Ghent University Hospital, Ghent, Belgium; 10 Data Science Institute, Ghent University Hospital, Ghent, Belgium; 11 Department of Anesthesiology and Reanimation, Cerrahpasa School of Medicine, Istanbul University-Cerrahpasa, Istanbul, Turkey; 12 3rd Department of Critical Care, “EVGENIDIO” Hospital, National and Kapodistrian University of Athens, Athens, Greece; 13 Department of General, Visceral and Thoracic Surgery, Klinikum Hannoversch-Muenden, Goettingen University, Göttingen, Germany; 14 Division of Scientific Affairs-Research, European Society of Intensive Care Medicine, Brussels, Belgium; 15 Anesthesia and Intensive Care Department, University Hospital of Modena, Modena, Italy; 16 UQ Centre for Clinical Research, Faculty of Medicine, The University of Queensland, Brisbane, QLD, Australia; 17 2nd Critical Care Department, Attikon University Hospital, Athens, Greece; 18 Department of Nursing, Faculty of Education, Health and Social Work, University College Ghent, Ghent, Belgium; 19 Jamieson Trauma Institute, The University of Queensland, Brisbane, QLD, Australia; 20 Nimes University Hospital, University of Montpellier, Nimes, France; 21 Critical Care Department, Hospital of the Interamerican Open University (UAI), Buenos Aires, Argentina; 22 Surgical Critical Care, Department of Anesthesia, Hospital Universitario La Paz-IdiPaz, Madrid, Spain; 23 Université de Paris, INSERM, UMR-S 1152-PHERE, Paris, France; 24 Anesthesiology and Critical Care Medicine, Bichat-Claude Bernard University Hospital, HUPNSV, AP-HP, Paris, France; 25 Faculty of Health Sciences, Poznan University of Medical Sciences, Poznan, Poland; 26 Department of Anaesthesiology and Intensive Therapy, Regional Hospital in Poznan, Poznan, Poland; 27 Intensive Care Department, Centro Hospitalar Universitario S. Joao, Faculty of Medicine, University of Porto, Grupo Infecao e Sepsis, Porto, Portugal; 28 Intensive Care Unit from Hospital Interzonal General de Agudos “Prof Dr Luis Guemes”, Buenos Aires, Argentina; 29 Ciberes and Vall d'Hebron Institute of Research, Barcelona, Spain; 30 Université Paris-Cité, IAME, INSERM 1137, Paris, France; 31 AP-HP, Hôpital Bichat, Medical and Infection Diseases ICU (MI2), Paris, France; 32 Department of General Internal Medicine and Infectious Diseases, AZ Delta, Roeselare, Belgium

**Keywords:** Pancreatic infection, Intensive care unit, Mortality, Intra-abdominal infection, Sepsis

## Abstract

**Background:**

The *AbSeS*-classification defines specific phenotypes of patients with intra-abdominal infection based on the (1) setting of infection onset (community-acquired, early onset, or late-onset hospital-acquired), (2) presence or absence of either localized or diffuse peritonitis, and (3) severity of disease expression (infection, sepsis, or septic shock). This classification system demonstrated reliable risk stratification in intensive care unit (ICU) patients with intra-abdominal infection. This study aimed to describe the epidemiology of ICU patients with pancreatic infection and assess the relationship between the components of the *AbSeS*-classification and mortality.

**Methods:**

This was a secondary analysis of an international observational study (“*AbSeS*”) investigating ICU patients with intra-abdominal infection. Only patients with pancreatic infection were included in this analysis (*n*=165). Mortality was defined as ICU mortality within 28 days of observation for patients discharged earlier from the ICU. Relationships with mortality were assessed using logistic regression analysis and reported as odds ratio (OR) and 95% confidence interval (CI).

**Results:**

The overall mortality was 35.2% (*n*=58). The independent risk factors for mortality included older age (OR=1.03, 95% CI: 1.0 to 1.1 *P*=0.023), localized peritonitis (OR=4.4, 95% CI: 1.4 to 13.9 *P*=0.011), and persistent signs of inflammation at day 7 (OR=9.5, 95% CI: 3.8 to 23.9, *P*<0.001) or after the implementation of additional source control interventions within the first week (OR=4.0, 95% CI: 1.3 to 12.2, *P*=0.013)*.* Gram-negative bacteria were most frequently isolated (*n*=58, 49.2%) without clinically relevant differences in microbial etiology between survivors and non-survivors.

**Conclusions:**

In pancreatic infection, a challenging source/damage control and ongoing pancreatic inflammation appear to be the strongest contributors to an unfavorable short-term outcome. In this limited series, essentials of the *AbSeS*-classification, such as the setting of infection onset, diffuse peritonitis, and severity of disease expression, were not associated with an increased mortality risk.

ClinicalTrials.gov number: NCT03270345

## Introduction

Acute pancreatitis is an acute abdominal condition which is self-limiting and uncomplicated in most cases of edematous pancreatitis. About 15%–30% of the cases develop more severe disease, leading to severe acute necrotic pancreatitis.^[^[1], [2], [3], [4]^]^ Severe acute necrotizing pancreatitis is characterized by high mortality due to a fulminant inflammatory process early in the course of the disease and the development of pancreatic and extra-pancreatic necrosis, infection, and multisystem organ failure (MOF) at a later stage.^[^^5^^]^ The characterization of these pathways has improved the understanding of the disease, leading to the identification of potential molecular targets, including the mechanisms of calcium signaling and acinar cell injury/death. Additionally, the protective role of the unfolded protein response and the toxic role of unsaturated fatty acids and has possible therapeutic implications. Further research, however, is needed to translate these insights into clinical benefits.^[^^6^^]^ Despite these achievements, the outcome of severe acute necrotizing pancreatitis has not improved significantly over the past years.^[^^2^^]^ Among other serious intra-abdominal infections, pancreatic infection complicating severe acute pancreatitis frequently poses a challenge in the intensive care unit (ICU) setting.^[^^7^^]^

For this group of patients, multidisciplinary and well-organized management is crucial, including close monitoring, appropriate use of imaging, proper fluid resuscitation, and use of antibiotics, nutritional and organ support. Additionally, the appropriate type and timing of endoscopic procedures, radiological interventions, and surgical interventions are critical.^[8]^ Surgical and interventional endoscopic source control are crucial and are both therapeutic and diagnostic. These interventions may include debridement of necrotic tissues (including transgastric necrosectomy), drainage of abscesses, control of the source of infection, restoration of anatomy and function, and, although infrequently performed, abdominal cleansing and irrigation. Often several surgical interventions are required at different stages of the disease.^[^^9^^,^^10^^]^

While a dysregulated immune response, together with hemodynamic and coagulation abnormalities, may lead to early onset organ failure (within 24–72 h), a second deterioration phase may be triggered by bacterial or fungal superinfection of the necrotic tissue (i.e., infected pancreatic necrosis), and may, together with exacerbation of the pre-existing inflammation, result in late-onset organ failure (beyond 2 weeks).^[^^2^^,^^11^^]^ Infection may be suspected in patients with a new, otherwise unexplained fever, (peri)pancreatic gas on imaging, bacteremia or candidemia in addition to worsening or persistent leukocytosis and other inflammatory parameters, and overall clinical deterioration.^[^^8^^,^^12^^]^ Bacterial superinfections occur in 10%–50% of patients with pancreatic necrosis.^[^[13], [14], [15], [16]^]^ Compared with sterile necrosis of the pancreas, pancreatic infection has a much worse prognosis, with mortality rates between 30% and 70%.^[^^8^^,^[17], [18], [19], [20], [21], [22], [23], [24], [25]^]^

As in any intra-abdominal infection, the microorganisms involved include a broad spectrum of Gram-positive and Gram-negative bacteria, as well as anaerobic bacteria and in pancreatic infection fungi. Multidrug-resistant (MDR) bacteria are more commonly observed in patients with recent exposure to broad-spectrum antibiotics, underlying medical conditions, and prior hospitalization for more than 5 days.^[^^10^^,^^26^^]^ In addition to patient-related factors, the risk of infection with MDR pathogens differs by the geographical region, local (in-hospital) ecology, and timing of appearance – depending on the disease severity and the intervention methods.^[^^10^^,^^27^^]^

Given the broad spectrum of presentation, the appropriate classification of intra-abdominal infection and sepsis has long been debated. The international *AbSeS* study aimed to demonstrate the relationship between a risk classification tool and mortality, independent of the type of intra-abdominal infection.^[^^7^^]^ The classification is based on three generic features of infection leading to specific clinical phenotypes of intra-abdominal infection. These features include (1) the setting of infection acquisition defined as either community, healthcare-associated, or early onset hospital-acquired (i.e., ≤7 days of hospital admission), or late-onset hospital-acquired infection (>7 days); (2) the absence or presence of anatomical barrier disruption, resulting either in localized or diffuse peritonitis;^[^^7^^,^^28^^]^ and (3) the severity of the disease manifestation, defined according to the Sepsis-3 criteria (i.e., infection, sepsis, or septic shock).^[^^29^^]^ The components of the *AbSeS*-classification were independently associated with mortality in the entire cohort, including all patients with intra-abdominal infection in the ICU and in the subgroup of patients with secondary peritonitis.^[^^7^^,^^30^^]^

Although the *AbSeS*-classification allowed adequate risk adjustment of different types of intra-abdominal infections, such as secondary peritonitis, its risk prediction value in other specific types of intra-abdominal infections remains uncertain. We aimed to assess the predictive value of the components of the *AbSeS-*classification in ICU patients with pancreatic infection. Therefore, the aim of this study was to assess risk factors for mortality in a subset of critically ill patients with pancreatic infection using the *AbSeS* risk classification.

## Methods

### Study design

**Figure 1:**
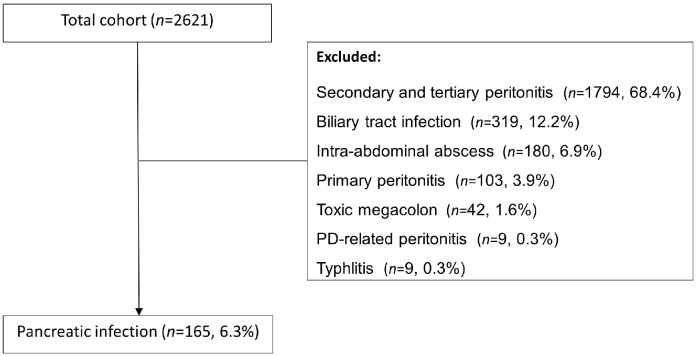
Included study population of pancreatic infection based on the total *AbSeS* study cohort.

This is a secondary analysis of data from the *AbSeS* study: a multinational observational cohort study of ICU patients with intra-abdominal infection.^[^^7^^]^ The *AbSeS* cohort included 2621 patients from 309 ICUs in 42 countries. For the present study, only patients with pancreatic infection were considered (*n*=165, Figure 1). Approval was granted by established national, regional, or local institutional review boards. The study is registered at ClinicalTrials.gov (NCT03270345). More detailed descriptions of inclusion and exclusion criteria, definitions, methods, and collection of data are described elsewhere.^[^^7^^]^

### Variables and definitions

Pancreatic infection was defined according to the International Sepsis Forum Consensus Conference on Definitions of Infection in the Intensive Care Unit.^[^^31^^]^ Patients were eligible for inclusion if they had (1) microbiologically confirmed pancreatic infection (i.e., positive cultures directly from the pancreas or the surrounding structures by percutaneous aspiration or direct visualization and culture at the time of surgery or from the bloodstream), (2) probable infected pancreatitis (i.e., the presence of surgical or radiographic evidence of an abnormal collection of an inflammatory focus within the substance of the pancreas or the surrounding structures with a positive Gram stain from the pancreatic collection without evidence from culture), or (3) possible infected pancreatitis (i.e., radiographic or direct surgical inspection with evidence suggestive of pancreatic abscess or other types of infection originating from the pancreas).

The following data were selected from the *AbSeS* database: demographics (sex, age, and body mass index [BMI]), comorbidities (cardiovascular disorders, chronic pulmonary disease, neurologic disease, diabetes, acquired immunodeficiency syndrome [AIDS], liver disease, chronic renal failure, immunosuppression, malnutrition, and obesity), lifestyle risk factors (tobacco, drug, and alcohol abuse), risk factors for MDR pathogens (nursing home resident, out-of-hospital parenteral/nutrition or vascular access, chronic dialysis, recent hospitalization, and antibiotics use in the past 6 months), and components of the *AbSeS*-classification as mentioned above.

The severity of acute illness was assessed using the Simplified Acute Physiology Score (SAPS II score) at the time of ICU admission ^[^^32^^]^ and the Sequential Organ Failure Assessment (SOFA score) at the time of diagnosis.^[^^33^^]^ The *AbSeS* protocol included a source control evaluation on day 7. Source control failure was defined as either the presence of persistent inflammation or the need for an additional intervention following the initial source control approach. However, source control evaluation is challenging in pancreatic infection. Persistent inflammation may be a sign of ongoing pancreatitis, although the infection is well-controlled. Additionally, recurrent interventions may be a strategy of damage control in acute infected pancreatitis as removal of necrotic material is often not done in a single procedure and can, therefore, not be used as a proxy of source control failure. Consequently, we decided to keep the clinical evaluation on day 7 in the study analysis but did not define it as either successful or failed source control. The clinical evaluation on day 7 was then categorized as “*stable*,” “*unstable as evidenced by persistent signs of inflammation*,” or “*unstable with need for recurrent interventions*.” In this evaluation, having “*persistent signs of inflammation*” may reflect either source control failure or ongoing pancreatitis. Likewise, the “*need for recurrent interventions*” may reflect either source control failure or a complicated case with the need for a stepwise damage control strategy. For patients who died before day 7, the source control evaluation was carried out on the last day alive.

Outcome data included ICU length of stay and ICU mortality within 28 days of observation for patients discharged earlier from the ICU.

To assess microbial etiology and cultures derived from intraoperative sampling, trans-abdominal fine-needle aspiration, abdominal drains (≤24 h post-surgery), and blood cultures were considered. The basic empiric antimicrobial coverage (targeting Gram-positive, Gram-negative or anaerobic bacteria, and fungi) was evaluated. EUCAST breakpoints are used as antimicrobial resistance thresholds.^[^^34^^]^ Antimicrobial resistance was defined as extended-spectrum beta-lactamase production (ESBL), carbapenem-resistance, and fluoroquinolone-resistance in Gram-negative microorganisms ^[^^35^^]^ and methicillin-resistance for *Staphylococcus aureus* (MRSA) or vancomycin-resistance in enterococci (VRE) for Gram-positive bacteria.

### Statistical analyses

Continuous variables were described with median values and 25th to 75th percentiles (interquartile range [IQR]), and frequency and percentage (%) were used to describe categorical variables. The Mann–Whitney *U* test was used for comparisons between continuous variables, and the chi-squared or Fisher's exact tests were used to compare categorical variables.

Logistic regression analysis was performed to assess relationships with mortality. The following variables were considered in the model (irrespective of their relationship in univariate analysis): demographics, comorbidities, day-7 clinical evaluation, and elements of the *AbSeS-*classification (i.e., setting of infection acquisition, anatomical disruption, and the severity of disease expression). Feature selection was performed using the stepwise approach to remove covariates with *P* >0.15. The final model contained, preferably, a maximum of one independent covariate per ten outcome events (dead). Results are reported as odds ratio (OR) and 95% confidence interval (CI). The SOFA score was not considered as it substantially overlaps with the severity of disease expression in the *AbSeS-*classification. Survival analysis of patients according to source control outcome was assessed using the Kaplan–Meier method, and the log-rank test was used to compare the survival curves. Statistical significance was defined as *P* <0.05. Statistical analyses were performed using SPSS Statistics version 28, and all tests were two-tailed.

## Results

### Description of the cohort

The study included 165 patients with pancreatic infection, with a mean age of 58 years. Samples for microbiological analysis were taken in 118 cases, with positive cultures in 89 patients (75.4%), hence considered proven cases of pancreatic infection (53.9%). Other cases were considered possible pancreatic necrosis (*n*=76, 46.1%) as no microbiological samples were taken (*n*=47, 28.5%) or results of Gram-staining were not reported (*n*=29, 17.6%). For patients who had their microbiological samples taken (*n*=118), Gram-negative bacteria were most frequently isolated (*n*=58, 49.2%): *Enterobacterales* were isolated in 50 patients (42.4%) and non-fermenting bacteria in 13 patients (11.0%). Gram-positive bacteria were isolated in 34 patients (28.8%), with enterococci being the most prevalent (*E. faecalis: n*=13, 11.0%; *E. faecium: n*=8, 6.8%). Anaerobic bacteria were isolated in 18 patients (15.3%) and *Candida* spp. in 16 patients (13.6%). MDR organisms were isolated in seven patients (5.9%).

The overall mortality was 35.2% (*n*=58). Table 1 summarizes patients’ characteristics for survivors and non-survivors. Overall, underlying conditions and lifestyle risk factors were more frequent in the non-survivor group. Non-survivors were older, had higher SOFA scores, and experienced more septic shock compared to survivors. Peritonitis (either localized or diffuse) and failure of source control were more common among non-survivors. The non-survivors presented more commonly with late-onset hospital-acquired infection. No difference between the groups was observed regarding the ICU length of stay. No clinically relevant differences in microbial etiology were observed between survivors and non-survivors.

**Table 1:** Characteristics of ICU patients with pancreatic infection according to survival status.

Characteristic	Survivors (*n*=107)	Non-survivors (*n*=58)	*P*-value
Demographics			
Age (years)	55 (41–69)	62 (53–74)	0.018
Sex, male	73/106 (68.2)	35/57 (61.4)	0.380
Type of ICU admission			0.556
Medical	56 (53.8)	27 (47.4)	
Surgical, non-emergency	10 (9.6)	8 (14)	
Surgical, emergency	38 (36.5)	22 (38.6)	
ICU stay (days)	15 (5–25)	16 (6–27)	0.717
Underlying conditions			
Chronic pulmonary disease	12 (11.2)	3 (5.2)	0.197
Malignancy	8 (7.5)	4 (6.9)	>0.999
Neurologic disease	2 (1.9)	4 (6.9)	0.186
Peptic ulcer disease	7 (6.5)	4 (6.9)	>0.999
Liver disease	4 (3.7)	5 (8.6)	0.280
Chronic renal failure	5 (4.7)	4 (6.9)	0.721
Myocardial infarction	5 (4.7)	7 (12.1)	0.115
Chronic heart failure			NA
Congestive heart failure	1 (0.9)	3 (5.2)	
Heart failure	0	3 (5.2)	
Peripheral vascular disease	4 (3.7)	2 (3.4)	>0.999
Diabetes mellitus	22 (20.6)	15 (25.9)	0.436
Immunosuppression	6 (5.6)	4 (6.9)	0.740
Lifestyle risk factors			
Malnutrition (body mass index <20 kg/m^2^)	3 (2.8)	4 (6.9)	0.213
Obesity (body mass index ≥30 kg/m^2^)	34 (31.8)	20 (34.5)	0.723
Tobacco use (>20 pack years)	18 (16.8)	9 (15.5)	0.829
Alcohol abuse (>10 g alcohol/day)	27 (25.2)	11 (19.0)	0.361
Severity of acute illness			
SAPS II score at the time of ICU admission	52 (39–60)	53 (40–65)	0.324
SOFA score at diagnosis	6.1 (4–8)	8.7 (5.8–12)	<0.001
*AbSeS-*classification			
Severity of disease expression			<0.001
Infection without sepsis	6 (5.6)	2 (3.4)	
Sepsis	75 (70.1)	23 (39.7)	
Septic shock	26 (24.3)	33 (56.9)	
Anatomical disruption			0.014
Not present	80 (74.8)	31 (53.4)	
Yes, with localized peritonitis	9 (8.4)	12 (20.7)	
Yes, with diffuse peritonitis	18 (16.8)	15 (25.9)	
Origin of infection onset			0.705
Community-acquired infection	30 (28.0)	15 (25.9)	
Early onset hospital-acquired infection (≤7 days)	23 (21.5)	10 (17.2)	
Late-onset hospital-acquired infection (≥7 days)	54 (50.5)	33 (56.9)	
Empiric antibiotic therapy	73 (76.0)	46 (90.2)	0.038

Continuous variables are presented as median (interquartile range). Categorical variables are presented as *n* (%).

ICU: Intensive care unit; NA: Not applicable; SAPS: Simplified Acute Physiology Score; SOFA: Sequential Organ Failure Assessment.

### Source control approaches

Data on source control are reported in Table 2. Source control interventions were performed in 138 patients (83.6%), including drainage (*n*=119, 72.1%), decompressive surgery (*n*=10, 6.1%), or restoration of anatomy and function (*n*=42, 25.5%). Drainage consisted of either open surgical drainage (*n*=96), laparoscopic drainage (*n*=3), peritoneal lavage (*n*=36), percutaneous drains (*n*=25), or debridement of necrosis (*n*=22). In certain cases, a combination of interventions was applied.

**Table 2:** Source control characteristics and clinical outcomes at day 7.

Items	Survivors (*n*=107)	Non-survivors (*n*=58)	*P*-value
Source control approach			
Initial source control applied	86 (80.4)	52 (89.7)	0.124
All drainage	72 (67.3)	47 (81.0)	0.060
Surgical drainage	57 (53.3)	39 (67.2)	0.082
Laparoscopic drainage	0	3 (5.2)	NA
Peritoneal lavage	20 (18.7)	16 (27.6)	0.187
Percutaneous drains	14 (13.1)	11 (19.0)	0.314
Debridement of necrosis	17 (15.9)	5 (8.6)	0.190
Decompressive surgery	9 (8.4)	1 (1.7)	0.101
Restoration of anatomy and function	28 (26.2)	14 (24.1)	0.775
Reasons for additional re-intervention			
Leakage	9 (8.4)	5 (8.6)	>0.999
Obstruction	2 (1.9)	1 (1.7)	>0.999
Abdominal compartment syndrome	1 (0.9)	1 (1.7)	>0.999
Bleeding	1 (0.9)	3 (5.2)	0.125
Ischemia/necrosis	3 (2.8)	1 (1.7)	>0.999
Abscess	0	0	NA
Explorative laparotomy for persistent inflammation	1 (0.9)	1 (1.7)	>0.999
Other	0 (0.0)	2 (3.4)	NA
Clinical evaluation at day 7, outcomes			<0.001
Stable	67 (62.6)	14 (24.1)	
Unstable, persistent signs of inflammation	24 (22.4)	32 (55.2)	
Unstable, additional intervention required following initial approach	16 (15.0)	12 (20.7)	

NA: not applicable.

### Clinical evaluation on day 7

On day 7, 81 cases (49.1%) were considered “stable,” 56 cases had persistent signs of inflammation (33.9%), and multiple source/damage control interventions were performed in 28 cases (17.0%). Reasons for re-intervention included leakage (*n*=14), obstruction (*n*=3), abdominal compartment syndrome (*n*=2), bleeding (*n*=4), ischemia/necrosis (*n*=4), explorative laparotomy for persistent inflammation (*n*=2), or others (*n*=2).

### Independent relationships with mortality

The objective of the logistic regression analysis was to assess relationships between the *AbSeS*-classification and mortality, alongside other risk factors for death. For that reason, we planned to “force” the *AbSeS*-classification components in the model irrespective of its relationship with the outcome. Due to the limited number of deceased patients (*n*=58), however, the final logistic regression model could not contain more than six independent covariates. The setting of the infection onset had no association with mortality. Therefore, this component of the *AbSeS*-classification was excluded from the model. The final model of the logistic regression analysis identified localized peritonitis, older age, and a clinical evaluation on day 7, indicating persistent inflammation or the need for repeated interventions for source/damage control as independent risk factors for death in critically ill patients with infected pancreatitis (Table 3). Other parameters, such as microbial etiology, comorbidities, and lifestyle risk factors, were not associated with mortality and were excluded (*P* >0.15).

**Table 3:** Independent relationships with mortality in critically ill patients with pancreatic infection.

Risk factor	OR	95%CI	*P-*value
Age (years increase)	1.0	1.0 to 1.1	0.023
Clinical evaluation on day 7 of the pancreatic infection process			
Stable	Reference		
Unstable, persistent signs of inflammation	9.5	3.8 to 23.9	<0.001
Unstable, additional intervention required following the initial approach	4.0	1.3 to 12.2	0.013
Anatomical disruption			
Not present	Reference		
Localized peritonitis	4.4	1.4 to 13.9	0.011
Diffuse peritonitis	1.8	0.7 to 4.6	0.201
Severity of disease expression			
Infection	Reference		
Sepsis	0.5	0.1 to 3.5	0.454
Septic shock	2.4	0.3 to 18.8	0.395

CI: confidence interval; OR: odds ratio.

**Figure 2:**
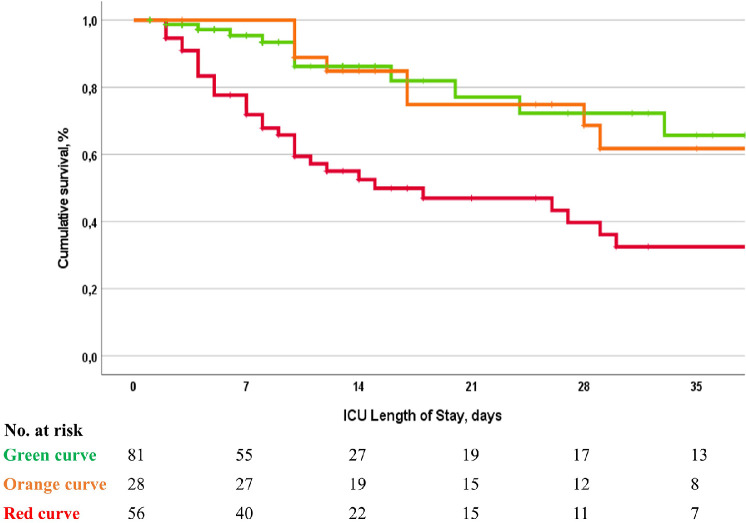
Survival curves according to clinical status at day 7 in critically ill patients with pancreatic infection. The green line represents a stable clinical status (i.e., without overt intra-abdominal inflammation or the need for additional interventions for either source control or damage control). These patients had a median age of 59 years (44–72). In this group, 58 patients had no anatomical barrier disruption (71.6%), 10 had localized peritonitis (12.3%), and 13 had diffuse peritonitis (16.0%). The orange line represents the need for additional intervention following the initial approach. These patients had a median age of 60 years (46–71). In this group, 16 patients had no anatomical barrier disruption (57.1%), 3 had localized peritonitis (10.7%), and 9 had diffuse peritonitis (32.1%). The red line represents a condition with persistent signs of inflammation. These patients had a median age of 59 years (48–75). In this group, 37 patients had no anatomical barrier disruption (66.1%), 8 had localized peritonitis (14.3%), and 11 had diffuse peritonitis (19.6%). The *P*-value for the Log-rank test for differences between the curves was <0.001.

Because of the significant relationship between the intermittent evaluation on day 7 and the outcome, we further analyzed the survival path stratified by the clinical status one week after diagnosis. These unadjusted survival curves are shown in Figure 2. The cumulative survival curve for a clinical condition with persistent signs of inflammation is significantly lower, with overall higher mortality.

## Discussion

This secondary analysis of the *AbSeS* study aimed to explore the association between mortality and independent risk factors in ICU patients with pancreatic infection. Our study is the first to report the specific implementation of the *AbSeS-*classification components for grading intra-abdominal infection in ICU patients with pancreatic infection. Mortality in ICU patients with pancreatic infection was high (35.2%), significantly greater than the overall mortality in the *AbSeS* cohort (i.e., 29.1%).^[^^7^^]^ The main finding of this study is that older age, localized peritonitis, and unstable clinical condition on day 7 (either due to persistent inflammation or the need for recurrent surgical intervention) are independently associated with mortality. The *AbSeS*-classification components, with previously recognized risk factors, such as late-onset hospital-acquired infection, diffuse peritonitis, and sepsis or septic shock, appeared to have no predictive value in this specific population.

In infected pancreatitis, localized peritonitis was associated with mortality, whereas diffuse peritonitis was not. This observation is counterintuitive, and this may be because diffuse peritonitis is rare in the cohort. Pancreatic infection rarely results in diffuse peritonitis being a retroperitoneal disease. The association with localized peritonitis should also be interpreted with caution as well due to the small numbers involved. A clinical picture of diffuse peritonitis may trigger earlier surgical intervention with a positive prognostic impact: studies have shown that delayed intervention is beneficial.^[^^8^^,^^12^^,^[36], [37], [38], [39], [40]^]^ In this regard, it is challenging to assess the timing of surgical intervention as the onset of infection on top of a chronic inflammatory process is impossible to determine.

Based on the entire cohort of the *AbSeS* study, Blot et al.^[^^7^^]^ identified the following variables as independent risk factors for mortality in intra-abdominal infections: higher SOFA scores, diffuse peritonitis, sepsis and septic shock, older age, malnutrition, diabetes mellitus, liver failure, and congestive heart failure. With the exception of older age, these aspects reflect the severity of the acute illness. In contrast, in the present study, the severity of acute illness and chronic underlying conditions did not prove to be significant determinants of mortality. Again, the limited study power must be considered, as it is highly uncommon for clinical aspects such as septic shock not to be associated with an increased risk of mortality. This association has also been reported by Zhu et al.^[^^41^^]^ in the specific context of patients with severe acute pancreatitis, albeit the outcome window of 24 h may have boosted the relative importance of septic shock as a risk factor for death.

These findings reiterate that adequate source control is the cornerstone in the management of pancreatic infection in ICU patients.^[^^7^^,^^16^^,^^24^^,^^39^^]^ A minimally invasive step-up approach is preferable, starting with the least invasive technique and gradually escalating in case of treatment failure.^[^^23^^,^^40^^,^^42^^,^^43^^]^ The present data indicate that recurrent interventions are associated with poor outcomes. However, this probably reflects complicated cases requiring multiple procedures in the pursuit of damage control. Invasive procedures also carry a risk of superinfection and may worsen the prognosis in patients with sterile necrosis.^[^^24^^]^ In pancreatic infections, the risk of superinfection is inferior to the beneficial impact of source control – antimicrobials as a single treatment appear to contribute less to infection control, primarily due to the persistence of infection in the necrotic tissues.

Delaying an intervention in stable patients is commonly recommended to allow for the development of a fibrous wall around the necrosis tissue.^[^^8^^,^^12^^,^[36], [37], [38], [39], [40]^]^ This strategy is associated with fewer complications, such as iatrogenic intraoperative injury, bleeding, postoperative septic and systemic inflammatory response, and reduced mortality.^[^^36^^]^ Earlier debridement is associated with higher mortality,^[^^8^^,^^12^^,^^16^^,^^44^^]^ whereas longer delay in intervention may imply overuse of antibiotics, leading to increased incidence of resistant bacteria and the emergence of fungi and other opportunistic microorganisms, all associated with prolonged ICU stay and increased mortality.^[^[45], [46], [47]^]^

Contrary to our conclusion, other studies conclude that conservative treatment with antibiotics as a single approach can resolve pancreatic infections and prevent the need for surgical intervention ^[^[48], [49], [50], [51], [52]^]^ or even reduce the risk of mortality.^[^^53^^,^^54^^]^ Few antibiotics (e.g., carbapenems, quinolones, and metronidazole) are believed to penetrate sufficiently into the necrotic infected focus.^[^^8^^,^^12^^]^ Tian et al.^[^^55^^]^ suggested restricting the use of broad-spectrum antibiotics and proposed more targeted drug administration based on specimen collection. For most of our patients, a conservative approach based on antimicrobials and organ support alone was probably not possible, given the high prevalence of sepsis or septic shock (Table 1).

Microbiologically, the spectrum of pathogenic microorganisms was generally monomicrobial, with a predominance of Gram-negative bacteria (35.2%), consistent with previous studies.^[^^7^^,^^10^^,^^55^^]^ While positive microbiology is as common as in the entire *AbSeS* cohort (71.5% and 75.6%, respectively), the *AbSeS* study reports a strikingly higher number of Gram-negative bacteria (58.6%) from clinical samples. Noor et al.^[^^56^^]^ claimed that there is a shift from Gram-negative to Gram-positive bacteria caused by the progression of pancreatitis and the longer hospital stay. Interestingly, a low number of patients (*n*=7, 4.2%) had MDR bacteria. The overall prevalence of antimicrobial resistance in the *AbSeS* study was higher (26.3%),^[^^7^^]^ which may be explained by a high incidence of nosocomial infections, especially late-onset hospital-acquired infections in an exclusively ICU cohort.^[^^55^^]^ Other studies also described the rising emergence of MDR bacteria.^[^^7^^,^^27^^,^^57^^,^^58^^]^ An explanation for the low MDR rate present in our cohort may be the current policy of withholding the initial empiric broad-spectrum antibiotics, despite ongoing inflammation and organ failure, until the most appropriate antimicrobial therapy is available based on culture results later in the course of the disease, with a secondary deterioration due to documented or probable infection. With this strategy, infection will develop without prior antimicrobial selective pressure toward MDR pathogens.

Infection of necrotic pancreatic tissue increases mortality and the probability of MOF, which is also an important determinant of death.^[^^18^^,^^20^^,^^22^^,^^25^^,^^39^^,^[59], [60], [61]^]^ The co-existence of both complications has a synergistic effect that doubles the risk of death.^[^^22^^,^^24^^]^ These determinants and the need for ICU admission represent the most severe forms of the disease.^[^^62^^]^ As our cohort exclusively contains ICU patients with pancreatic infection, they are considered at high risk for extra-pancreatic complications.^[^^5^^,^^55^^]^

As previously shown, age has a significant impact on mortality in acute pancreatitis.^[^^63^^]^ Similar to the study by Tian et al.,^[^^55^^]^ the mean age of patients in the non-survival group was significantly higher than in the survival group. Compared to the overall cohort of the *AbSeS* study, the mean age in the present cohort is significantly lower (66 years and 58 years, respectively), indicating that acute pancreatitis in the ICU is associated with high mortality rates irrespective of age.

First, per protocol, the *AbSeS* database only included patients with infection, making it impossible to assess the impact of infection in a subset of patients with acute pancreatitis. Second, some observations are counterintuitive, indicating that our study might be underpowered. In fact, with only 58 deceased patients, the number of covariates allowed in a logistic regression model to avoid overfitting is quite low. Therefore, larger studies are necessary to elucidate further the impact of the variables in the *AbSeS-*classification on mortality in ICU patients with pancreatic infection. Third, variables potentially influencing the outcome might not have been collected (e.g., the etiology of pancreatitis and location and extent of necrosis).^[^^64^^]^ Fourth, no specific data were collected regarding the classification of acute pancreatitis, according to the revised Atlanta classification by Banks et al.^[^^65^^]^ Yet, we assume that the included cases had severe pancreatic infections according to the definitions of the study protocol. Fifth, acute pancreatitis is a complicated disease, as it involves triggering a severe inflammatory process, distinguishing it from other intra-abdominal infections. Therefore, these cases are more difficult to categorize or predict with the *AbSeS*-classification, especially concerning the setting of the infection onset, where the majority are late complications: in contrast to secondary peritonitis, where the infection is part of the disease process from the beginning. Furthermore, the timing of surgical intervention as from infection onset proved impossible as it is unclear when the case of acute pancreatitis became complicated with infection. Finally, as already outlined in the methods section, in infected pancreatitis, source control evaluation on day 7 as per the *AbSeS* protocol proved unusable.

## Conclusions

Older age, localized peritonitis, and a clinical condition reflecting persistent inflammation at 7 days, as well as the need for recurrent interventions within a week in pursuit of source or damage control, were the strongest predictors of mortality in critically ill patients with pancreatic infection. The *AbSeS-*classification system did not predict mortality in this sample of patients.

## Author Contributions

Marie Dejonckheere: Writing original draft preparation, statistical analysis, investigation. Massimo Antonelli: conceptualization, data collection, investigation, review and editing. Kostoula Arvaniti: data collection, investigation, review and editing. Koen Blot: database management, investigation, review and editing. Ben Creagh-Brown: data collection, investigation, review and editing. Dylan W. de Lange: data collection, investigation, review and editing. Jan De Waele: conceptualization, data collection, investigation, review and editing. Mieke Deschepper: database management and statistical analysis (advanced), investigation, review and editing. Yalim Dikmen: data collection, investigation, review and editing. George Dimopoulos: conceptualization, data collection, investigation, review and editing. Christian Eckmann: conceptualization, data collection, investigation, review and editing. Guy Francois: conceptualization, project administration, interpretation data, review and editing. Massimo Girardis: data collection, investigation, review and editing. Despoina Koulenti: conceptualization, data collection, investigation, review and editing. Sonia Labeau: project administration, database management, investigation, review and editing. Jeffrey Lipman: conceptualization, data collection, investigation, review and editing. Fernando Lipovestky: data collection, investigation, review and editing. Emilio Maseda: data collection, investigation, review and editing. Philippe Montravers: conceptualization, data collection, investigation, review and editing. Adam Mikstacki: data collection, investigation, review and editing. Jose-Artur Paiva: conceptualization, data collection, investigation, review and editing. Cecilia Pereyra: data collection, investigation, review and editing. Jordi Rello: conceptualization, data collection, investigation, review and editing. Jean-Francois Timsit: conceptualization, data collection, investigation, review and editing. Dirk Vogelaers: conceptualization, supervision, data collection, investigation, review and editing. Stijn Blot: funding acquisition, conceptualization, supervision, project administration, data collection, investigation, original draft preparation.

## Acknowledgements

The study is registered at ClinicalTrials.gov (number NCT03270345) prior to conducting the research.

## Funding

*AbSeS* is a Trials Group Study of the European Society of Intensive Care Medicine and was supported by a Pfizer investigator-initiated research grant. Received grants related to the submitted work: S. Blot (Pfizer). Received honoraria or grants outside the submitted work: M. Antonelli (Fresenius, Pfizer, Toray); J. De Waele (Research Foundation Flanders, Pfizer, Bayer, MSD); C. Eckmann (Merck, Pfizer); J. Lipman (MSD, Pfizer); E. Maseda (Astellas Pharma, Pfizer, MSD).

## Ethics Statement

Not applicable.

## Conflict of Interest

The authors declare that they have no known competing financial interests or personal relationships that could have appeared to influence the work reported in this paper.

## AbSeS is a Trials Group Study of the European Society of Intensive Care Medicine (ESICM)

**Collaborators AbSeS Study**

**National coordinators**

**Algeria:** Amin Lamrous (CHU Alger), **Argentina:** Cecilia Pereyra (Hospital Interzonal Agudos Prof Dr Luis Guemes, Buenos Aires), Fernando Lipovestky (Universidad Abierta Interamericana Hospital, Buenos Aires); **Australia**: Despoina Koulenti (UQCCR, Faculty of Medicine, The University of Queensland, Brisbane); **Belgium**: Jan De Waele (Ghent University Hospital, Ghent); **Canada**: Joao Rezende-Neto (St Michael's Hospital, Toronto); **Colombia**: Yenny Cardenas (Hospital Universitario Fundación Santa Fe, Bogotá); **Czech Republic**: Tomas Vymazal (Motol University Hospital, Prague); **Denmark**: Hans Fjeldsoee-Nielsen (Nykoebing Falster Hospital, Nykoebing Falster); **France**: Philippe Montravers (CHU Bichat Claude Bernard, Paris); **Germany**: Matthias Lindner (geb. Kott) (Universitätsklinikum, Schleswig-Holstein, Kiel); **Greece**: Arvaniti Kostoula (Papageorgiou General Hospital, Thessaloniki); **India**: Yash Javeri (Nayati Healthcare, Delhi); **Italy**: Massimo Girardis (University Hospital of Modena, Modena); **Israel**: Sharon Einav (Shaare Zedek Medical Centre, Jerusalem); **Netherlands**: Dylan de Lange (University Medical Center, Utrecht); **Peru**: Luis Daniel Umezawa Makikado (Clínica Ricardo Palma, Lima); **Poland**: Adam Mikstacki (Regional Hospital, Poznan); **Portugal**: José-Artur Paiva (Centro Hospitalar UniversitárioSao João, Porto); **Romania**: Dana Tomescu (Fundeni Clinical Institute, Bucharest); **Russian Federation**: Alexey Gritsan (Krasnoyarsk State Medical University, Krasnoyarsk Regional Clinical Hospital, Krasnoryarsk); **Serbia**; Bojan Jovanovic (Clinical Center of Serbia, Belgrade); **Singapore**: Kumaresh Venkatesan (Khoo Teck Puat Hospital, Singapore); **Slovenia**: Tomislav Mirkovic (University Medical Centre, Ljubljana); **Spain**: Emilio Maseda (Hospital Universitario La Paz, Madrid); **Turkey**: Yalim Dikmen (Istanbul University-Cerrahpasa, Cerrahpasa Medical School, Istanbul); **United Kingdom**: Benedict Creagh-Brown (Royal Surrey County Hospital NHS Foundation Trust, Guilford).

***Investigators***

**Algeria**: CHU (Algiers): Amin Lamrous.

**Argentina:** Sanatorio Güemes (Buenos Aires): Monica Emmerich, Mariana Canale; Sanatorio de la Trinidad Mitre (Buenos Aires): Lorena Silvina Dietz, Santiago Ilutovich; Hospital General de Agudos “Dr. Teodoro Alvarez” (Buenos Aires): John Thomas Sanchez Miñope, Ramona Baldomera Silva; Hospital Militar Central (Buenos Aires): Martin Alexis Montenegro, Patricio Martin; Policlinico Central Union Obrera Metalurgica (Buenos Aires): Pablo Saul, Viviana Chediack; Sanatorio San José (Buenos Aires): Giselle Sutton, Rocio Couce; Hospital General de Agudos “Dr. Ignacio Pirovano” (Buenos Aires): Carina Balasini, Susana Gonzalez; Hospital Britanico (Buenos Aires): Florencia Maria Lascar, Emiliano Jorge Descotte; CMPF Churruca-Visca (Buenos Aires): Natalia Soledad Gumiela, Carina Alejandra Pino; Clinica San Camilo (Buenos Aires): Cristian Cesio, Emanuel Valgolio; Hospital Francisco Javier Muñiz (Buenos Aires): Eleonora Cunto, Cecilia Dominguez; Universidad Abierta Interamericana Hospital (Buenos Aires): Fernando Lipovestky; Hospital Alberto Balestrini (Buenos Aires): Nydia Funes Nelson, Esteban Martin Abegao; Hospital Interzonal Agudos Prof Dr Luis Güemes (Buenos Aires): Cecilia Pereyra, Norberto Christian Pozo; Hospital Español (Buenos Aires): Luciana Bianchi, Enrique Correger; Clinica Zabala (Caba): Maria Laura Pastorino, Erica Aurora Miyazaki; Hospital César Milstein (Caba): Norberto Christian Pozo, Nicolas Grubissich; Hospital Regional Victor Sanguinetti (Comodoro): Mariel Garcia, Natalia Bonetto; Hospital Municipal de Urgencias (Cordoba): Noelia Elizabeth Quevedo, Cristina Delia Gomez; Hospital Manuel B Cabrera (Coronel Pringles): Felipe Queti, Luis Gonzalez Estevarena; Hospital Español de Mendoza (Mendoza), Godoy Cruz: Ruben Fernandez, Ignacio Santolaya; H.I.G.A. Prof. Dr. Luis Güemes (Haedo): Norberto Christian Pozo; Hospital Municipa Doctor Carlos Macias (Mar de Ajo): Sergio Hugo Grangeat, Juan Doglia; Hospital Luis C. Lagomaggiore (Mendoza): Graciela Zakalik, Carlos Pellegrini; Hospital Nacional Profesor Alejandro Posadas (Moron): Maria Monserrat Lloria, Mercedes Esteban Chacon; Hospital Provincial de Neuquen (Neuquen): Mariela Fumale; Clinica Modelo S.A (Paraná): Mariela Leguizamon; Sanatorio de la Ciudad (Puerto Madryn): Irene Beatriz Hidalgo, Roberto Juli*an*Tiranti; Sanatorio Nosti (Rafaela): Paola Capponi, Agustin Tita; Hospital Provincial del Centenario (Rosario): Luis Cardonnet, Lisandro Bettini; Sanatorio Parque (Rosario): Agñel Ramos, Luciano Lovesio; Hospital Papa Francisco (Salta): Edith Miriam Miranda, Angelica Beatriz Farfan; Hospital San Juan Bautista (San Fernando del Valle de Catamarca): Carina Tolosa, Lise Segura; Hospital Central San Isidro Dr Melchor A. Posse (San Isidro-Buenos Aires): Adelina Bellocchio, Brian Alvarez; Hospital Guillermo Rawson (San Juan): Adriana Manzur, Rodolfo Lujan; EstablecimientoAsistencial Dr Lucio Molas (Santa Rosa): Natalia Fernandez, Nahuel Scarone; Clínica de Especialidades (Villa María): Alan Zazu, Carina Groh.

**Australia:** The Bendigo Hospital (Bendigo): Jason Fletcher, Julie Smith; Coffs Harbour Health Campus (Coffs Harbour): Raman Azad, Nitin Chavan; Concord Hospital (Concord): Helen Wong; Mark Kol; Royal Darwin Hospital (Darwin): Lewis Campbell; Royal Brisbane and Women's Hospital (Herston, Brisbane): Despoina Koulenti, Therese Starr; Sir Charles Gairdner Hospital (Nedlands): Brigit Roberts, Bradley Wibrow; Redcliffe Hospital (Redcliffe): Timothy Warhurst; St Vincent's Hospital (Toowommba): Meher Chinthamuneedi, Bernal Buitrago Ferney.

**Belgium:** Cliniques du Sud Luxembourg (CSL)-Hôpital Saint-Joseph (Arlon): Marc Simon; Chirec Hospital (Braine-l'Alleud): Daniel De Backer; Cliniques Universitaires St Luc (Brussels): Xavier Wittebole; Brugmann University Hospital (Brussels): David De Bels, Cliniques de l'Europe – St-Michel (Brussels): Vincent Collin; University Hospital Antwerp (Edegem): Karolien Dams, Philippe Jorens; Ghent University Hospital (Ghent): Jan De Waele, Mieke Deschepper; University College Ghent (Ghent): Sonia Labeau; Jessa Ziekenhuis (Hasselt): Jasperina Dubois; University Hospitals Leuven (Leuven): Jan Gunst; CHU Ambroise Paré (Mons): Lionel Haentjens; Clinique Saint-Pierre (Ottignies): Nicolas De Schryver, Thierry Dugernier.

**Canada**: St. Michael's Hospital (Toronto): Joao Rezende-Neto, Sandro Rizoli.

**Chile**: Hospital Clinico Viña del Mar (Viña del Mar): Paul Santillan.

**China**: Jiangsu Province Hospital (Nanjing): Yi Han; Yangpu Hospital of Tongji University (Shanghai): Ewelina Biskup, Changjing Qu; Urumqi General Hospital (Urumqi): Xinyu Li, Wannan Medical College First Affiliated Hospital, Yijishan Hospital (Wuhu): Tao Yu, Lu Weihua.

**Colombia**: Clinica Universitaria Colombia (Bogota): Daniel Molano-Franco, José Rojas, Mederi Hospital (Bogota): Juan Mauricio Pardo Oviedo; Dario Pinilla; Hospital Universitario Fundación Santa Fe (Bogota): Yenny Cardenas, Edgar Celis; Clinica Santa Gracia (Popayan): Mario Arias.

**Croatia**: Opća bolnica Dubrovnik (Dubrovnik): Anita Vukovic, Maja Vudrag; General Hospital Karlovac (Karlovac): Matija Belavic, Josip Zunic; Clinical Hospital Center Rijeka (Rijeka): Janja Kuharic, Irena Bozanic Kricka; University Hospital Center of Zagreb (Zagreb): Ina Filipovic-Grcic, Boris Tomasevic; University Hospital Center Sestre Milosrdnice (Zagreb): Melanija Obraz, Bruna Bodulica.

**Czech Republic**: Nemocnice Břeclav (Břeclav): Martin Dohnal; University Hospital Brno (Brno): Jan Malaska, Milan Kratochvil; Municipal Hospital (Havirov): Igor Satinsky, Peter Schwarz; Hospital Karlovy Vary (Karlovy Vary): Zdenek Kos; University Hospital Olomouc (Olomouc): Ladislav Blahut; University Hospital of Ostrava (Ostrava): Jan Maca; Institute for Clinical and Experimental Medicine (Prague): Marek Protus, Eva Kieslichová.

**Denmark**: Odense University Hospital (Odense): Louise Gramstrup Nielsen, Birgitte Marianne Krogh.

**Ecuador**: San Vicente de Paúl Hospital (Ibarra): Francisco Rivadeneira; Hospital Oncologico “Dr. Julio Villacreses Colmont” SOLCA (Portoviejo): Freddy Morales, José Mora; Hospital General Puyo (Puyo): Alexandra Saraguro Orozco; Hospital de Especialidades “Eugenio Espejo” (Quito): Diego Rolando MorochoTutillo, Nelson Remache Vargas; Clinica La Merced (Quito): Estuardo Salgado Yepez; Hospital Militar (Quito): Boris Villamagua.

**Egypt**: Kasr El AINI Hospital, Cairo University (Cairo): Adel Alsisi, Abdelraouf Fahmy.

**France**: CHU Amiens (Amiens): Hervé Dupont; CHU Angers (Angers): Sigismond Lasocki; Hôpital Beaujon (Clichy): Catherine Paugam-Burtz, Arnaud Foucrier; Centre Hospitalier Compiegne Noyon (Compiègne): Alexandru Nica, Geneviève Barjon; Centre Hospitalier de Lens (Lens): Jihad Mallat; Hôpital Edouard Herriot (Lyon): Guillaume Marcotte; Hôpital Nord (Marseille): Marc Leone, Gary Duclos; Clinique du Millénaire (Montpellier): Philippe Burtin; CHU Bichat Claude Bernard (Paris): Philippe Montravers, Enora Atchade; Groupe Hospitalier Paris Saint-Joseph (Paris): Yazine Mahjoub, Benoît Misset; Hôpital Bichat (Paris): Jean-François Timsit, Claire Dupuis; CHU de Rouen, Hôpital Charles Nicolle (Rouen): Benoît Veber; Centre Hospitalier Yves le Foll (Saint-Brieuc): Matthieu Debarre; Hôpitaux Universitaires de Strasbourg, NHC – Nouvel Hôpital Civil (Strasbourg): Oliver Collange; Hôpitaux Universitaires de Strasbourg, Hôpital de Hautepierre (Strasbourg): Julien Pottecher, Stephane Hecketsweiler; Hôpital Cochin (Paris): Mélanie Fromentin, Antoine Tesnière.

**Germany**: University Hospital Giessen (Giessen): Christian Koch, Michael Sander; Universitätsklinikum Schleswig-Holstein (Kiel): Matthias Kott, Gunnar Elke; University Hospital of Leipzig (Leipzig): Hermann Wrigge, Philipp Simon.

**Greece**: General Hospital of Agios Nikolaos (Agios Nikolados): Anthoula Chalkiadaki, Charalampos Tzanidakis; Democritus University of Thrace (Alexandroupolis): Ioannis Pneumatikos, Eleni Sertaridou; Evangelismos Hospital (Athens): Zafiria Mastora, Ioannis Pantazopoulos; Hippocrateion General Hospital of Athens (Athens): Metaxia Papanikolaou, Theonymfi Papavasilopoulou; General Hospital Laiko (Athens): John Floros, Virginia Kolonia; University Hospital Attikon (Athens): George Dimopoulos, Chryssa Diakaki; General Hospital Asklepieio Voulas (Athens): Michael Rallis, Alexandra Paridou; General Hospital G. Gennimatas (Athens): Alexandros Kalogeromitros, Vasiliki Romanou; Konstantopouleio Hospital (Athens): Charikleia Nikolaou, Katerina Kounougeri; Agioi Anargiroi General Oncological Hospital of Kifissia (Athens): Evdoxia Tsigou, Vasiliki Psallida; Red Cross Hospital (Athens): Niki Karampela, Konstantinos Mandragos; General Hospital St George (Chania): Eftychia Kontoudaki, Alexandra Pentheroudaki; Thriassio General Hospital of Eleusis (Eleusis): Christos Farazi-Chongouki, Agathi Karakosta; Giannitsa General Hospital (Giannitsa): Isaac Chouris, Vasiliki Radu; University Hospital Heraklion (Heraklion): Polychronis Malliotakis, Sofia Kokkini; Venizelio General Hospital of Heraklion (Heraklion): Eliana Charalambous, Aikaterini Kyritsi; University Hospital of Ioannina (Ioannina): Vasilios Koulouras, Georgios Papathanakos; General Hospital Kavala (Kavala): Eva Nagky, Clairi Lampiri; Lamia General Hospital (Lamia): Fotios Tsimpoukas, Ioannis Sarakatsanos; Agios Andrea's General Hospital of Patras (Patras): Panagiotis Georgakopoulos, Ifigeneia Ravani; Tzaneio General Hospital (Pireaus): Athanasios Prekates, Konstantinos Sakellaridis; General Hospital of Pyrgos (Pyrgos Hleias): Christos Christopoulos, Efstratia Vrettou; General Hospotal of Rethymnon (Rethymnon): Konstantinos Stokkos, Anastasia Pentari; Papageorgiou Hospital (Thessaloniki): Kostoula Arvaniti, Kyriaki Marmanidou; Hippokration Hospial (Thessaloniki): Christina Kydona, Georgios Tsoumaropoulos; G. Papanikolaou General Hospital (Thessaloniki): Militisa Bitzani, Paschalina Kontou; Agios Pavlos Hospital (Thessaloniki): Antonios Voudouris, Elli-Nikki, Flioni; General Hospital of Thessaloniki G.Gennimatas (Thessaloniki): Elli Antypa, Eleftheria Chasou; Theagenio Anticancer Hospital (Thessaloniki): Souzana Anisoglou, Eirini Papageorgiou; General Hospital of Trikala (Trikala): Theoniki Paraforou, Agoritsa Tsioka; Achillopoyleio General Hospital Volos (Volos): Antigoni Karathanou; Xanthi General Hospital (Xanthi): Aristeidis Vakalos.

**India**: CIMS Hospital (Ahmedabad): Bhagyesh Shah, Chirag Thakkar; CHL Hospitals (Indore): Nikhilesh Jain; Sanjay Gandhi Postgraduate Institute of Medical Sciences (SGPGIMS) (Lucknow): Mohan Gurjar, Arvind Baronia; Ruby Hall Clinic (Pune): Prachee Sathe, Shilpa Kulkarni; Jubilee Mission Medical College & Research Institute (Thrissur): Cherish Paul, John Paul.

**Iran**: Nemazi Hospital (Shiraz): Mansoor Masjedi; Anesthesiology and Critical Care Research Center, Shiraz University of Medical Sciences (Shiraz): Reza Nikandish, Farid Zand; Shiraz Trauma Hospital (Shiraz): Golnar Sabetian; Shohada Hospital (Tabriz): Ata Mahmoodpoor; Masih Daneshvari Hospital (NRITLD) (Tehran): Seyed Mohammadreza Hashemian.

**Israel**: Hadassah Hebrew University Medical Center (Jerusalem): Miklosh Bala.

**Italy**: Cardarelli Ospedale (Campobasso): Romeo Flocco, Sergio Torrente; PinetaGrande Private Hospital (Castel Volturno): Vincenzo Pota; Arcispedale Sant'Anna (Ferrara): Savino Spadaro, Carlo Volta; University Hospital of Modena (Modena): Massimo Girardis, Giulia Serafini; Ospedale S.Antonio (Padova): Sabrina Boraso, Ivo Tiberio; Azienda Ospedaliera Universitaria Policlinico Paolo Giaccone (Palermo): Andrea Cortegiani, Giovanni Misseri; Azienda Ospedaliero-Universitaria di Parma (Parma): Maria Barbagallo, Davide Nicolotti; Azienda Ospedaliero-Universitaria Pisana (Pisa): Francesco Forfori, Francesco Corradi; Fondazione Policlinico Universitario A.Gemelli IRCCS (Roma): Massimo Antonelli, Gennaro De Pascale; Regina Elena National Cancer Institute of Rome (Roma): Lorella Pelagalli; Azienda Ospedaliero-Universitaria Citta della Salute e della Scienza di Torino, Presidio Ospedaliero Molinette (Torino): Luca Brazzi, Ferdinando Giorgio Vittone; Policlinico Universitario GB Rossi (Verona): Alessandro Russo, Davide Simion; University-Hospital of Foggia (Foggia): Antonella Cotoia, Gilda Cinnella.

**Jamaica**: University Hospital of the West Indies (Kingston): Patrick Toppin, Roxanne Johnson-Jackson.

**Japan**: Kameda General Hospital (Kamogawa): Yoshiro Hayashi, Ryohei Yamamoto; Japanese Red Cross Musashino Hospital (Tokyo): Hideto Yasuda, Yuki Kishihara; Okinawa Prectural Chube Hospital (Uruma, Okinawa): Junji Shiotsuka.

**Mexico**: UMAE Hospital Especialidades Antonio Fraga Mouret-Centro Medico Nacional La Raza IMSS (Mexico City): Luis Alejandro Sanchez-Hurtado, Brigitte Tejeda-Huezo; Hospital Juárez de Mexico (Mexico City): Luis Gorordo; Instituto Nacional de Cancerologia (Mexico City): Silvio A. Ñamendys-Silva, Francisco J. Garcia-Guillen; Hospital general # 5 IMSS (Nogales, Sonora): Manuel Martinez; Hospital Regional de Alta Especialidad de la Península de Yucatán (Merida, Yacatan): Erick Romero-Meja, Ever Colorado-Dominguez.

**Netherlands**: Deventer Hospital (Deventer): Huub van den Oever, Karel Martijn Kalff; Medisch Spectrum Twente (Enschede): Wytze Vermeijden, Alexander Daniel Cornet; Tjongerschans Hospital (Heerenveen): Oliver Beck, Nedim Cimic; Zuyderland Medisch Centrum (Heerlen): Tom Dormans, Laura Bormans; Erasmus MC University Medical Center (Rotterdam): Jan Bakker, Ditty Van Duijn; Elisabeth-TweeSteden Ziekenhuis (Tilburg): Gerrit Bosman, Piet Vos; University Medical Center (Utrecht): Dylan de Lange, Jozef Kesecioglu; Diakonessenhuis (Utrecht): Lenneke Haas.

**Oman**: Khoula Hospital (Muscat): Akram Henein.

**Paraguay**: Hospital Regional de Luque (Luque): Ariel M. Miranda.

**Peru**: Clínica Ricardo Palma (Lima): Luis Daniel Umezawa Makikado, Gonzalo Ernesto Gianella Malca; Victor Lazarte Echegaray Hospital (Trujillo): Abel Arroyo-Sanchez.

**Poland**: Silesian Hospital Cieszyn (Cieszyn): Agnieszka Misiewska-Kaczur; Wojewodzki Szpital Zesoloby w Koninie (Konin): Frisch Akinyi; First Public Teaching Hospital (Lublin): Miroslaw Czuczwar; Szpital Wojewodzki w Opolu SPZOZ (Opole): Karolina Luczak; SPZZOZ w Ostrowi Mazowieckiej (Ostrow Mazowiecka): Wiktor Sulkowski; Poznan University of Medical Sciences, Regional Hospital in Poznan (Poznan): Barbara Tamowicz, Adam Mikstacki; Centrum Medyczne (Poznan): Beata Swit, Bronisław Baranowski; University Hospital (Poznan): Piotr Smuszkiewicz, Iwona Trojanowska; WSM im. J. Strusia (Poznan): Stanislaw Rzymski; Niepubliczny Zakład Opieki Zdrowotnej Szpital w Puszczykowie im. prof. Stefana Tytusa Dąbrowskiego (Puszczykowo): Mariusz Sawinski, Marta Trosiak; Infant Jesus Teaching Hospital of Warsaw Medical University (Warsaw): Malgorzata Mikaszewska-Sokolewicz.

**Portugal**: Hospital de Braga (Braga): Ricardo Alves, Dina Leal; Centro Hospitalar Algarve (Faro): Andriy Krystopchuk, Pedro Muguel Hilario Mendonca; Centro Hospitalar Universit**á**rio Lisboa Central – Hospital Curry Cabral (Lisboa): Rui Antunes Pereira; Centro Hospitalar Universit**á**rio Lisboa Norte – Hospital de Santa Maria (Lisboa): Maria Raquel Lopes Marques de Carvalho, Carlos Candeias; Hospital Pedro Hispano (Matosinhos): Elena Molinos, Amélia Ferreira; Centro Hospitalar Sao Joao – Serviço Medicina Intensiva – UCIPU (Porto): Guiomar Castro, José-Artur Paiva; Centro Hospitalar Sao Joao – Serviço Medicina Intensiva – UCIPG (Porto): José-Manuel Pereira; Centro Hospitalar Sao Joao – Infectious Diseases ICU (Porto): Lurdes Santos, Alcina Ferreira; Hospital do Litoral Alentejano (Santiago do Cacém): Dulce Pascoalinho; São Bernardo – Centro Hospitalar Setubal (Setubal): Rosa Ribeiro, Guilherme Domingos; Hospital Vila Franca de Xira (Vila Franca de Xira): Pedro Gomes, David Nora; Centro Hospitalar de Trás-os-Montes e Alto Douro (Vila Real): Rui Pedro Costa, Anabela Santos.

**Qatar**: Hamad Medical Corporation (Doha): Ahmed Subhy Alsheikhly.

**Romania**: Fundeni Clinical Institute (Bucharest): Dana Tomescu, Mihai Popescu; Regional Institute of Oncology (lasi): Ioana Grigoras, Emilia Patrascanu.

**Russian Federation**: Krasnodar Regional Hospital #2 (Krasnodar): Igor Zabolotskikh, Tatiana Musaeva; Krasnoyarsk State Medical University, Krasnoyarsk Regional Clinical Hospital (Krasnoyarsk): Alexey Gritsan, Denis Gaigolnik; Vishnevsky Institute of Surgery (Moscow): Vladimir Kulabukhov; Privolzhskiy District Medical Center (Nizhniy Novgorod): Vladislav Belskiy; Clinical Hospital # 4 (Perm): Nadezhda Zubareva, Maxim Tribulev.

**Saudi Arabia**: International Medical Center (Jeddah): Ahmed Abdelsalam, Ayman Aldarsani; King Faisal Specialist Hospital & Research Centre (Riyadh): Muhammad Al-Khalid; PSMMC (Riyadh): Ghaleb Almekhlafi, Yasser Mandourah.

**Serbia**: Clinical Centre of Serbia (Belgrade): Bojan Jovanovic, Krstina Doklestic; Clinic for Digestive Surgery (Belgrade): Jelena Velickovic, Dejan Velickovic; Clinical Center Nis, (Nis): Radmilo Jankovic, Anita Vukovic; Oncology Institute of Vojvodina (Sremska Kamenica): Svetlana Skoric-Jokic, Dragana Radovanovic.

**South Africa**: Charlotte Maxeke Johannesburg Academic Hospital (Johannesburg): Guy Richards, Ahmad Alli.

**Spain**: Complejo Hospitalario Universitario de Albacete (Albacete): Maria del Carmen Cordoba Nielfa, Rafael Sánchez Iniesta; Parc de Salut Mar (Barcelona): Adela Benítez-Cano Martínez, Carlos Garcia Bernedo; Hospital Delfos (Barcelona): Santiago Alberto Picos Gil; Vall d'Hebron University Hospital (Barcelona): Xavier Nuvials, Jordi Rello; Hospital Universitario de Basurto (Bilbao): Joseba Gonzalez Garcia, Jose Manuel Garcia Peña; Hospital General Universitario Santa Lucia (Cartagena): Roberto Jimenez, Luis Herrera; Hospital General Universitari de Castelló (Castelló): Laura Galarza Barrachina, Ignacio Catalan Monzon; Hospital General Universitario de Ciudad Real (Ciudad Real): Francisco Javier Redondo, Ruben Villazala; Hospital Costa de la Luz (Huelva): Diego Fernando Matallana Zapata, Isabel Maria Villa Lopez; Hospital Universitari de Bellvitge (L'Hospitalet de Llobregat): Gabriel Moreno-Gonzalez, Juan Carlos Lopez-Delgado; Hospital Universitario de Canarias (La Laguna): Jorge Solera Marin; Hospital Universitario Severo Ochoa (Léganes): Purificacion Sanchez-Zamora; Hospital Universitari Arnau de Vilanova (Lleida): Montserrat Vallverdú Vidal; Hospital Quirón Campo de Gibraltar (Cádiz): Jesús Flores González; Hospital Universitarion del Henares (Madrid): Irene Salinas, Cecilia Hermosa; Hospital Universitario La Paz (Madrid): Emilio Maseda; Hospital Clinico San Carlos (Madrid): Fernando Martinez-Sagasti, Sara Domingo-Marín; Central de la Defensa Gomez Ulla (Madrid): Johanna Abril Victorino; Hospital 12 de Octubre (Madrid): Raquel Garcia-Alvarez, Pablo López-Arcas Calleja; Hospital Universitario de Malaga (Malaga): Maria-Victoria de la Torre-Prados; CHU Ourense (Ourense): Pablo Vidal-Cortes, Lorena del Río-Carbajo; Complejo Hospitalario de Navarra (Pamplona): Javier Izura, Victoria Minguez; Hospital Universitari Mutua Terrassa (Terrassa): Josep Trenado Alvarez, Anna Parera Prous; Complejo Hospitalario de Toledo (Toledo): Daniel Paz; Hospital Verge de la Cinta (Tortosa): Ferran Roche-Campo; Hospital Clínico Universitario de Valencia (Valencia): Gerardo Aguilar, Javier Belda; Rio Hortega University Hospital (Valladolid): Jesus Rico-Feijoo, Cesat Aldecoa; Hospital Clinico Universitario Lozano Blesa (Zaragoza): Begoña Zalba-Etayo.

**Switzerland**: Kantonsspital Frauenfeld (Frauenfeld): Martin Lang; Alexander Dullenkopf.

**Thailand**: Faculty of Medicine Vajira Hospital, Navamindradhiraj University (Bangkok): Konlawij Trongtrakul; Anusang Chtsomkasem.

**Turkey**: Düzce University Hospital (Duzce): Türkay Akbaş; Ankara University School of Medicine (Ankara): Mustafa Necmettin Unal, Menekse Ozcelik; Akdeniz University Medical School (Antalya): Ayca Gumus, Atilla Ramazanoglu; Trakya University Medical Faculty (Edirne): Dilek Memis, Inal Mehmet; Istanbul University-Cerrahpasa, Cerrahpasa Medical School (Istanbul): Yalim Dikmen, Seval Urkmez; Haydarpaşa Numune Training and Research Hospital (Istanbul): Asu Ozgultekin; Istanbul University Cerrahpasa Medical School Hospital (Istanbul): Oktay Demirkiran; Medipol Mega Hospitals Complex (Istanbul): Nesrin Ahu Aslan, Deniz Kizilaslan; Uludag University, School of Medicine (Nilüfer/Bursa): Ferda Kahveci, Nurdan Ünlü; Elazig Training & Research Hospital (Elazig): Zeynep Ozkan.

**United Kingdom**: Aberdeen Royal Infirmary (Aberdeen): Callum Kaye, Jan Jansen; Antrim Area Hospital (Antrim): Orla O'Neill, Christopher Nutt; Barnet General Hospital, RFL NHS FT (Barnet): Rajeev Jha, Nicolas Hooker; Basingsoke & North Hampshire Hospital (Basingstoke): Irina Grecu, Christina Petridou; Royal Victoria Hospital (Belfast): Murali Shyamsundar, Lia McNamee; Ulster Hospital (Belfast): John Trinder, Samantha Hagan; Belfast City Hospital (Belfast): Catriona Kelly, Jonathon Silversides; Brighton and Sussex University Hospitals (Brighton): Casiano Barrera Groba, Owen Boyd; West Suffolk Hospital NHS Foundation Trust (Bury St Edmunds): Kaushik Bhowmick, Sally Humphreys; Cambridge University Hospitals NHS Foundation Trust and University of Cambridge (Cambridge): Charlotte Summers, Petra Polgarova; Western Sussex NHS Foundation Trust, St Richard's Hospital (Chichester, West Sussex): Michael Margarson, Justin Dickens; Colchester General Hospital (Colchester): Suzanne Pearson, Elaine Chinery; Altnagelvin Hospital (Derry): Noel Hemmings, Sinead O'Kane; Ninewells Hospital (Dundee): Pauline Austin, Stephen Cole; Medway NHS Foundation Trust (Gillingham): Catherine Plowright, Roberta Box; Queen Elizabeth University Hospital (Glasgow): Christopher Wright, Lorna Young; Royal Surrey County Hospital (Guildford): Ben Creagh-Brown, Laura Montague; Aintree University Hospital (Liverpool): Robert Parker; Ben Morton; Guy's and St Thomas Hospitals (London): Marlies Ostermann, Julia Bilinska; University Hospital Lewisham (London): Bernd Oliver Rose, Rosie Reece-Anthony; St Georges University Hospitals NHS Foundation Trust (London): Christine Ryan, Mark Hamilton; King's College Hospital (London): Philip Hopkins, Julia Wendon; Luton and Dunstable Hospital (Luton): Giovanni Brescia, Nazia Ijaz; Maidstone and Tunbridge Wells NHS Trust Hospital (Maidstone): James Wood, Michelle George; Prince Charles Hospital (Merthyr Tydfil): Piroska Toth-Tarsoly; Northumbria Specialist Emergency Care Hospital (Newcastle Upon Tyne): Bryan Yates, Maureen Armstrong; Royal Victoria Infirmary (Newcastle Upon Tyne): Carmen Scott, Christine Boyd; Royal Gwent Hospital (Newport): Tamas Szakmany, David Rees; Kings Mill Hospital (Nottingham): Paul Pulak, Mandy Coggon; Royal Oldham Hospital (Oldham): Bhaskar Saha, Linda Kent; Royal Glamorgan Hospital (Pontyclun): Bethan Gibson; Poole Hospital NHS FT (Poole): Julie Camsooksai, Henrik Reschreiter; East Surrey Hospital (Redhill): Pat Morgan, Sivatharshini Sangaralingham; Conquest Hospital (St Leonards-on-sea): Alastair Lowe, Petr Vondras; Lister Hospital (Stevenage): Sunil Jamadarkhana, Carina Cruz; University Hospital of North Tees (Stockton-on-Tees): Rakesh Bhandary; Sunderland Royal Hospital (Sunderland): Peter Hersey, Julie Furneval; Musgrove Park Hospital (Taunton): Richard Innes, Patricia Doble; Warwick Hospital (Warwick): Ben Attwood, Penny Parsons; Watford General Hospital (Watford): Valerie Page, Xiaobei Zhao; Royal Hampshire County Hospital (Winchester): Irina Grecu, Julian Dalton.

**United Arab Emirates**: Sheikh Khalifa Medical City (Abu Dhabi): Mohammed Hegazy, Yasser Awad.

**United States**: Cleveland Clinic (Cleveland): Douglas Naylor, Amanda Naylor; Detroit Medical Center (Detroit): Sarah Lee; University of South Alabama Medical Center (Mobile, AL): Sidney Brevard, Noelle Davis.
